# Environmental circulation of multiple avian influenza subtypes and potential association with human infections in central China

**DOI:** 10.3389/fcimb.2026.1911586

**Published:** 2026-09-10

**Authors:** Bicong Wu, Sheng Zhao, Yifei Nie, Chunxiao Liu, Hong Bo, Xue Luo, Yun Song, Lin Zhu, Baifan Zhang, Yujiao Mu, Haiyan Wei, Jingjing Pan, Haifeng Wang, Ying Ye, Linqi Diao, Hongxia Ma

**Affiliations:** 1 Henan Key Laboratory of Infectious Pathogenic Microbiology, Henan Provincial Center for Disease Control and Prevention, Zhengzhou, Henan, China; 2 Henan Province Academy of Preventive Medicine, Zhengzhou, China; 3 School of Public Health, Henan University of Medicine, Xinxiang, Henan, China; 4 National Institute for Viral Disease Control and Prevention, Chinese Center for Disease Control and Prevention, Beijing, China

**Keywords:** Avian influenza virus, environmental surveillance, H3N8, H9N2, live poultry markets, one health, zoonotic transmission

## Abstract

**Introduction:**

Avian influenza viruses (AIVs) continue to circulate in live poultry markets (LPMs), posing potential risks for zoonotic exposure. This study investigated the environmental circulation of AIVs and their potential epidemiological associations with human infection in Henan Province, China.

**Methods:**

We analyzed 7,956 environmental samples collected from poultry-associated settings in Henan Province between 2020 and 2025. Multivariable logistic regression was used to identify factors associated with AIV detection. Serological surveillance was conducted among occupationally exposed individuals, and phylogenetic analyses were performed to characterize the genetic relationships between human-derived and environmental AIVs.

**Results:**

A total of 1,460 samples (18.35%) tested positive for AIVs. LPMs were significantly associated with higher odds of AIV detection (OR = 7.211, 95% CI: 5.039–10.319, P < 0.001), and the odds of AIV detection increased over the surveillance period (OR = 1.254, 95% CI: 1.210–1.301, P < 0.001). Multiple AIV subtypes were detected, with H9N2 remaining predominant throughout the study period. Five human infections were identified, including H5N6 and H3N8 infections in 2022 and three H9N2 infections in 2025. Serological surveillance identified H9N2-seropositive and H3N8-reactive samples among occupationally exposed individuals. Phylogenetic analyses revealed genetic similarities between human-derived and environmental viruses. Three H3N8 viruses, including one isolated from a human case, formed a distinct phylogenetic cluster, with their internal genes closely related to co-circulating H9N2 viruses.

**Discussion:**

These findings demonstrate sustained circulation of multiple AIV subtypes in poultry-associated environments and highlight the importance of integrated environmental and human surveillance for the early detection of viruses with zoonotic potential.

## 1 Introduction

Avian influenza viruses (AIVs) belong to the genus *Alphainfluenzavirus* and are classified into subtypes H1–H18 and N1–N11 based on hemagglutinin (HA) and neuraminidase (NA) combinations. These viruses infect a wide range of avian species and occasionally mammals (Bi et al., 2024; Subedi et al., 2024; Putra et al., 2025). As a transboundary animal disease caused by AIVs, avian influenza can spread across geographic regions and among host populations through poultry trade, migratory bird movements, and contaminated environments, posing continued risks to animal and public health (The Global Consortium for H5N8 and Related Influenza Viruses, 2016; McElwain and Thumbi, 2017; Bi et al., 2020; Blagodatski et al., 2021). Recent widespread outbreaks of H5N1 clade 2.3.4.4b, including documented spillover into dairy cattle in North America, have further illustrated the continuous expansion of AIV host ranges and strong ecological adaptability (Webby and Uyeki, 2024; Mohammad et al., 2026). These global changes in AIV ecology and host range highlight the need for continuous regional surveillance in areas with intensive poultry production and frequent human-poultry interactions, including central China, to monitor AIV diversity, evolution, and potential zoonotic risks.

Among these subtypes, H9N2 has become enzootic in poultry populations across Asia and is frequently detected in LPMs. Despite the implementation of vaccination programs, H9N2 viruses continue to circulate in poultry populations, highlighting the need for continuous veterinary surveillance to monitor viral evolution and antigenic changes (Liu et al., 2023; Pardini et al., 2026). Although infections in poultry are generally mild or asymptomatic, H9N2 viruses serve as gene donors for multiple reassortant influenza viruses. Internal gene segments derived from H9N2 have been identified in several emerging subtypes, including H7N9, H10N3, H10N5, and H3N8, highlighting the role of H9N2 in ongoing viral evolution within poultry populations (Wang et al., 2014; Yang et al., 2022; Yang et al., 2025c). In China, sporadic human H9N2 infections have been reported and are commonly associated with exposure to live poultry or poultry-related environments (Iacobucci, 2026; Zhang et al., 2026).

Henan Province, an important poultry production and trading region in central China, has reported several human infections with different AIV subtypes in recent years, while multiple AIV subtypes continue to circulate in poultry-associated environments. However, the long-term co-circulation dynamics of diverse AIV subtypes and potential associations with human cases within poultry-associated environments remain insufficiently characterized in this region. Existing regional surveillance data in Henan remain fragmented, and no previous study has systematically integrated multi-year environmental monitoring, serological surveys, human case tracing, and whole-genome sequencing to dissect the circulation patterns, genetic diversity, and potential zoonotic risks of H9N2, H3N8, and H5 viruses. This critical knowledge gap limits regional zoonotic risk assessment and the formulation of targeted prevention and control strategies under a One Health framework in central China (Liu et al., 2023; Bo et al., 2025). To address this knowledge gap, we conducted a six-year environmental surveillance study in Henan Province, China, integrating epidemiological, serological, and genomic analyses.

## 2 Materials and methods

### 2.1 Sample collection and detection

Environmental surveillance was conducted from January 2020 to December 2025 in Henan Province, China, as part of the national influenza monitoring network. The surveillance network covered 17 of the 18 prefecture-level cities in Henan Province for nearly full geographic coverage. Sampling sites were selected according to local poultry production and trading characteristics, geographic distribution, and surveillance feasibility. Due to variations in surveillance priorities and local conditions, monitoring sites and annual sampling intensity were not completely identical throughout the study period. Sampling sites included LPMs, backyard poultry flocks (BPFs), commercial poultry farms (CPFs), poultry slaughtering and processing plants (PSPs), and wild bird habitats (WBHs). A total of 72 surveillance sites were included, comprising 30 LPMs, 11 BPFs, 22 CPFs, 8 PSPs, and 1 WBH. The detailed distribution of surveillance sites across prefecture-level cities is provided in **Supplementary Table 1**.

Monthly environmental sampling was performed, and collected specimens encompassed poultry drinking water (PDW), feces (F), cage surface swabs (CSS), chopping board swabs (CBS), poultry washing wastewater (PWW), defeathering machine swabs (DMS), feathers (Fe), and eggshell swabs (ESS). Each specimen was suspended in phosphate-buffered saline (PBS) supplemented with antibiotics and cryopreserved at −70 °C pending testing.

Respiratory samples from laboratory-confirmed human cases were collected by local Centers for Disease Control and Prevention agencies following national surveillance protocols and sent to our laboratory for virological analysis.

Serological surveillance was conducted annually from October to March of the following year between 2020 and 2025 among occupationally exposed populations with direct exposure to poultry-associated environments (LPMs, BPFs, CPFs, PSPs, and WBHs) following the national avian influenza serological surveillance protocol. Blood specimens were tested for anti-AIV antibodies, and the targeted subtypes were selected according to national surveillance requirements and the predominant circulating AIV subtypes during each surveillance period. Participants were recruited from designated surveillance sites according to the national surveillance protocol and were enrolled based on predefined occupational exposure criteria and willingness to participate rather than through population-based random sampling. During the study period, serum samples were collected from 78 surveillance sites across 14 administrative regions in Henan Province. Blood samples were collected and stored at 4 °C overnight, followed by centrifugation at 2,000 rpm for 10 min. Separated sera were aliquoted and stored at −20 °C until testing.

Environmental samples and respiratory specimens from laboratory-confirmed human cases were screened for influenza A virus via real-time reverse transcription PCR (rRT-PCR). The testing was performed using the Influenza A Virus Nucleic Acid Detection Kit (Jiangsu Bioperfectus Technologies Co., Ltd., Taizhou, Jiangsu, China), following the manufacturer’s instructions. Samples with Ct values ≤ 37 were considered positive, whereas samples with Ct values > 40 or no amplification were considered negative. Samples with Ct values > 37 and ≤ 40 were retested according to the manufacturer’s criteria. Samples testing positive for influenza A were subjected to subtyping for H5, H7 and H9 avian influenza viruses with the Avian Influenza Virus H5/H7/H9 Subtype Nucleic Acid Detection Kit (Jiangsu Bioperfectus Technologies Co., Ltd., Taizhou, Jiangsu, China). Specimens negative for these three subtypes were further examined for other influenza A subtypes using the Influenza A Virus HxNy Rapid Subtyping Kit (Shanghai BioGerm Medical Technology Co., Ltd., Shanghai, China). For this kit, samples with Ct values ≤ 35 were considered positive, samples with Ct values > 38 or no amplification were considered negative, and samples with Ct values of 35–38 were retested according to the manufacturer’s instructions.

### 2.2 Serological assays

Hemagglutination inhibition (HI) and microneutralization (MN) assays were performed in accordance with the World Health Organization influenza laboratory diagnosis and surveillance manual (World Health Organization, 2011; Bo et al., 2026). Serum samples were treated with receptor-destroying enzyme, heat-inactivated, and serially diluted two-fold starting at 1:10. HI assays were performed using beta-propiolactone-inactivated viruses with turkey or chicken red blood cells, depending on the surveillance period and reference virus. MN assays were performed using 100 TCID_50_ of virus in MDCK cells, and MN titers were defined as the reciprocal of the highest serum dilution yielding at least 50% neutralization.

A/Henan/4-10CNIC/2022(H3N8) was used for H3N8 serological testing during 2021–2025, whereas H3N8 testing was not conducted during 2020–2021. For H9N2, A/Anhui-Lujiang/39/2018(H9N2), A/Guizhou/11495/2021(H9N2), and A/Anhui-Tianjian/11086/2022(H9N2) were used as reference viruses in different surveillance periods. Ferret antisera raised against these viruses were used as positive controls in the HI and MN assays and were provided by the Chinese National Influenza Center. Due to the absence of internationally established serological criteria for H3N8 viruses, samples with HI or MN titers of ≥ 10 were considered H3N8-reactive rather than seropositive. H9N2 seropositivity was defined as both HI and MN titers of 80 or higher.

### 2.3 Virus isolation

Virus isolation assays were implemented strictly as specified by the National Influenza Surveillance Technical Guidelines (2017 edition). Selected influenza A virus-positive environmental samples were subjected to virus isolation based on sample freshness and viral RNA abundance, with priority given to freshly collected samples with Ct values < 35. Samples were inoculated into the allantoic cavity of 10-day-old specific pathogen-free (SPF) embryonated chicken eggs and incubated at 37 °C for 48 h. Allantoic fluids were then harvested and tested for influenza A virus by real-time RT-PCR to confirm successful virus isolation.

### 2.4 Whole-genome sequencing and sequence assembly

Viral RNA was extracted from isolated virus strains using the QIAamp Viral RNA Mini Kit (QIAGEN, Germany) according to the manufacturer’s instructions. Whole-genome amplification was performed using the ULSEN^®^ Ultra-Sensitive Influenza A Virus Whole Genome Capture Kit (Beijing MicroFuture Biotechnology Co., Ltd., Beijing, China). Sequencing libraries were prepared from purified amplicons and sequenced on an Illumina MiSeq platform using a paired-end strategy.

Raw sequencing reads were processed using standard quality-control procedures, including adapter trimming and removal of low-quality reads. The remaining high-quality reads were assembled into complete genome segments using CLC Genomics Workbench (version 26). Genome completeness and sequencing quality were evaluated based on sequencing depth and genome coverage. The obtained consensus genomes showed complete genome coverage (100%) with high sequencing depth.

### 2.5 Sequence collection and quality control

Influenza A virus sequences of H3N8, H5N6, and H7N9 from China (2010–2025) were retrieved from GISAID, and H9N2 sequences (1994–2025) were obtained from GISAID and NCBI. These were combined with 40 sequences generated in this study (**Supplementary Table 2**). Sequences were aligned using MAFFT (version 7.505) and assessed with SeqKit (version 2.12.0). Low-quality sequences were removed based on genome completeness, coverage (< 95%), and sequencing errors, followed by manual inspection of the alignment prior to downstream analyses.

### 2.6 Molecular characterization and nucleotide difference analysis

Key amino acid substitutions associated with receptor binding, polymerase activity, mammalian adaptation, virulence, antigenic variation, and antiviral resistance were identified based on whole-genome sequence alignments. Sequence alignments and amino acid substitution analysis were performed using MEGA 12, and mutation frequencies were calculated among analyzed isolates. Functional annotations of identified substitutions were assigned based on previously published studies.

Pairwise nucleotide differences between the human-derived H9N2 virus and related environmental H9N2 viruses were calculated using a custom Python script (Python version 3.13) based on aligned whole-genome sequences. The number of nucleotide differences was summarized for each of the eight gene segments.

### 2.7 Phylogenetic and evolutionary analysis

Phylogenetic analyses of the eight gene segments of H3N8 viruses and the six internal gene segments of H9N2 viruses were performed using the maximum likelihood method implemented in IQ-TREE (version 2.0.7), and bootstrap values from 1,000 replicates were displayed at relevant nodes.

Time-scaled phylogenetic and evolutionary analyses of the HA and NA genes of H9N2 were conducted in BEAST (version 1.10.5) using a GTR+Γ4 substitution model, an uncorrelated lognormal relaxed molecular clock, and a coalescent constant-size prior, and posterior probabilities were shown at relevant nodes. The substitution model was used based on previous studies of H9N2 avian influenza virus evolution (Xia et al., 2022). The uncorrelated lognormal relaxed molecular clock was applied to account for potential rate variation among viral lineages. A coalescent constant-size prior was used as a simple demographic model because the dataset did not provide strong evidence supporting a specific population growth pattern. MCMC chains were run for 200 million steps with sampling every 20,000 steps. Convergence was assessed in Tracer (version 1.7.2), and all parameters achieved effective sample sizes (ESS) greater than 200 after a 10% burn-in. Maximum clade credibility trees were generated using TreeAnnotator (version 1.10.5) and visualized using FigTree (version 1.4.4) and the Interactive Tree of Life (iTOL).

### 2.8 Data analysis

Statistical analyses were performed using Python (version 3.13). Differences in positivity rates between groups were assessed using the chi-square (χ²) test, with *P* < 0.05 considered statistically significant. To identify factors independently associated with AIV positivity, multivariable logistic regression analysis was performed. AIV detection status (positive/negative) was used as the dependent variable, and sampling year, season, surveillance setting, and sample type were included as explanatory variables. Odds ratios (ORs) with 95% confidence intervals (CIs) were calculated, and *P* < 0.05 was considered statistically significant. Figures were generated using Matplotlib and R (version 4.5.1).

## 3 Results

### 3.1 Environmental epidemiological characteristics of AIV in Henan Province

**Figure 1:**
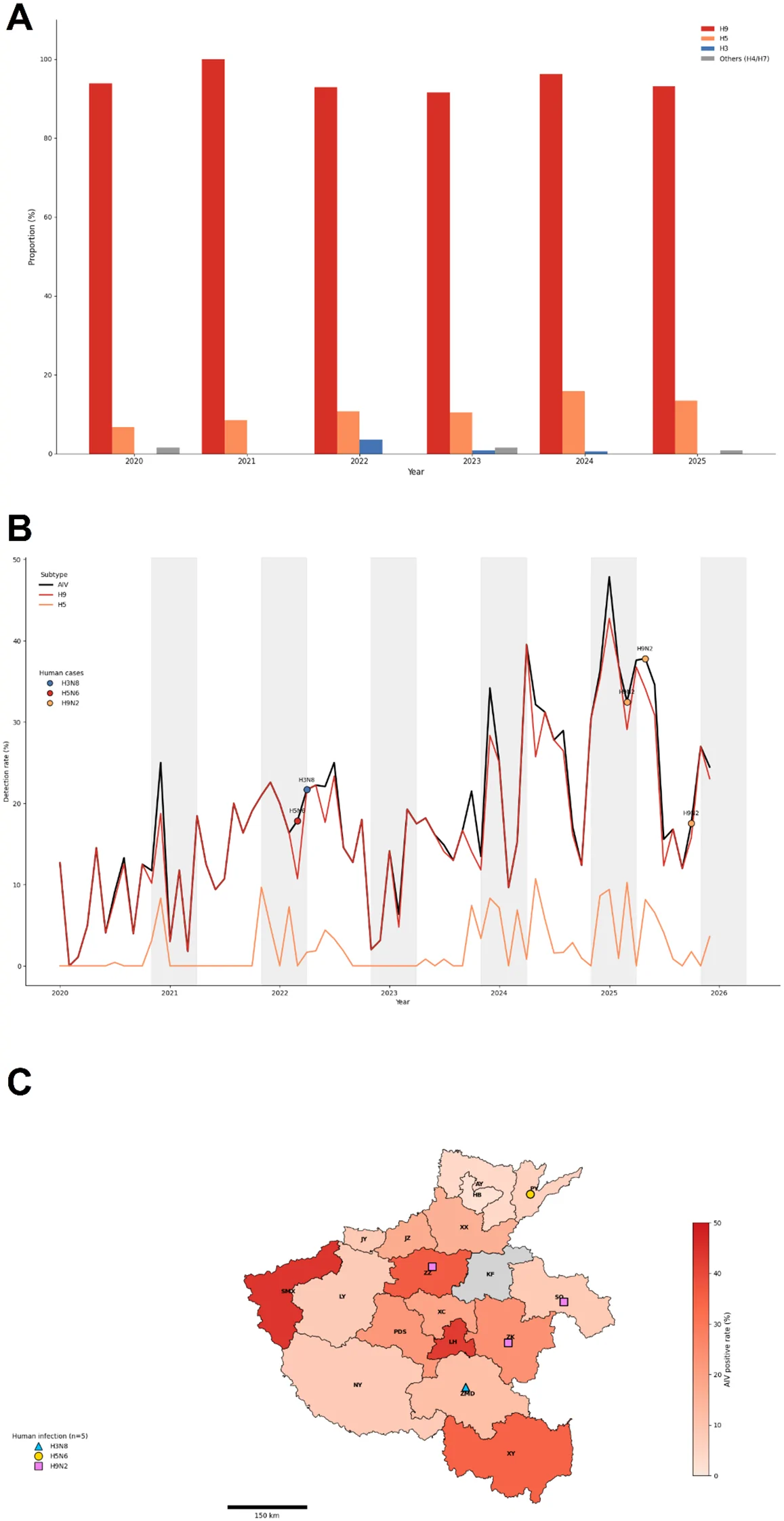
Spatiotemporal dynamics and subtype distribution of avian influenza viruses (AIVs) and human infections in Henan Province, China (2020–2025). **(A)** Annual proportions of AIV subtypes (H9, H5, H3, and others). **(B)** Monthly detection rates of overall AIVs and major subtypes H9 and H5. Shaded areas indicate the winter-spring season (November-March) and are provided for contextual reference only. Human infection cases (H3N8, H5N6, and H9N2) are marked. **(C)** Spatial distribution of AIV positivity rates across cities in Henan Province, classified into four levels (<10%, 10-20%, 20-30%, >30%), with human cases overlaid as star symbols.

From 2020 to 2025, 7,956 environmental samples were collected in Henan Province, among which 1,460 were positive for AIV (18.35%, 1,460/7,956). AIV detection rates differed substantially among surveillance settings, with LPMs showing the highest detection rate (29.41%, 1,345/4,574) and being significantly associated with increased odds of AIV detection in multivariable logistic regression analysis (OR = 7.211, 95% CI: 5.039–10.319, *P* < 0.001), suggesting that LPMs represent high-contact environments where AIV contamination is more frequently detected (**Table 1**). Among different sample types, DMS samples showed the highest odds of AIV detection (OR = 2.012, 95% CI: 1.547–2.617, *P* < 0.001). In addition, co-detection of multiple AIV subtypes was observed most frequently in DMS and PWW samples (**Supplementary Figure 1**). H9N2 remained the predominant subtype throughout the surveillance period, accounting for the majority of AIV-positive samples, whereas H5 and H3 viruses were detected sporadically (**Figure 1A**). Higher AIV detection rates were observed during the later years of surveillance, particularly 2024–2025 (**Figure 1B**). This temporal pattern was further supported by multivariable logistic regression, which identified sampling year as an independent predictor of AIV detection (OR = 1.254, 95% CI: 1.210–1.301, *P* < 0.001). Seasonal variation remained a significant predictor of AIV detection, with increased detection probabilities during winter (OR = 1.417, 95% CI: 1.182–1.700, *P* < 0.001) and spring (OR = 1.366, 95% CI: 1.145–1.629, *P* < 0.001) compared with autumn (**Table 1**). Spatial heterogeneity in AIV detection was evident across Henan Province (**Figure 1C**). Although human cases were identified in areas with sustained environmental AIV circulation, their geographic distribution was not completely concordant with the overall pattern of environmental detections.

**Table 1:** Environmental surveillance characteristics and multivariable logistic regression analysis of factors associated with AIV detection in Henan Province, China, 2020–2025.

Variable	Category	No. tested	No. positive	Positivity (%)	OR	95% CI	P value
Surveillance setting							
	BPFs	692	34	4.91	Reference	—	—
	CPFs	2,214	43	1.94	0.345	0.217–0.547	<0.001
	LPMs	4,574	1,345	29.41	7.211	5.039–10.319	<0.001
	PSPs	464	38	8.19	1.859	1.138–3.037	0.013
	WBHs	12	0	0	—	—	—
Sample type							
	CBS	960	241	25.1	Reference	—	—
	CSS	1,831	282	15.4	1.28	1.038–1.578	0.021
	DMS	405	152	37.53	2.012	1.547–2.617	<0.001
	ESS	274	11	4.01	0.246	0.130–0.467	<0.001
	F	1,750	323	18.46	0.941	0.770–1.149	0.55
	Fe	348	43	12.36	1.138	0.770–1.680	0.517
	PDW	1,071	130	12.14	1.219	0.940–1.582	0.136
	PWW	1,100	232	21.09	0.861	0.695–1.066	0.17
	Other	217	46	21.2	1.049	0.719–1.529	0.805
Season							
	Autumn	1,971	303	15.37	Reference	—	—
	Spring	2,063	416	20.16	1.366	1.145–1.629	<0.001
	Summer	2,155	374	17.36	1.14	0.954–1.361	0.15
	Winter	1,767	367	20.77	1.417	1.182–1.700	<0.001
Year	Per year increase	—	—	—	1.254	1.210–1.301	<0.001
Total	—	7,956	1,460	18.35	—	—	—

OR, odds ratio; CI, confidence interval; BPFs, backyard poultry flocks; CPFs, commercial poultry farms; LPMs, live poultry markets; PSPs, poultry slaughtering and processing plants; WBHs, wild bird habitats; CBS, cage bedding swabs; CSS, cage surface swabs; DMS, drinking water/material swabs; ESS, equipment surface swabs; PDW, poultry drinking water; PWW, poultry wastewater.

### 3.2 Characteristics of human AIV infections and associated environmental and serological findings

**Figure 2:**
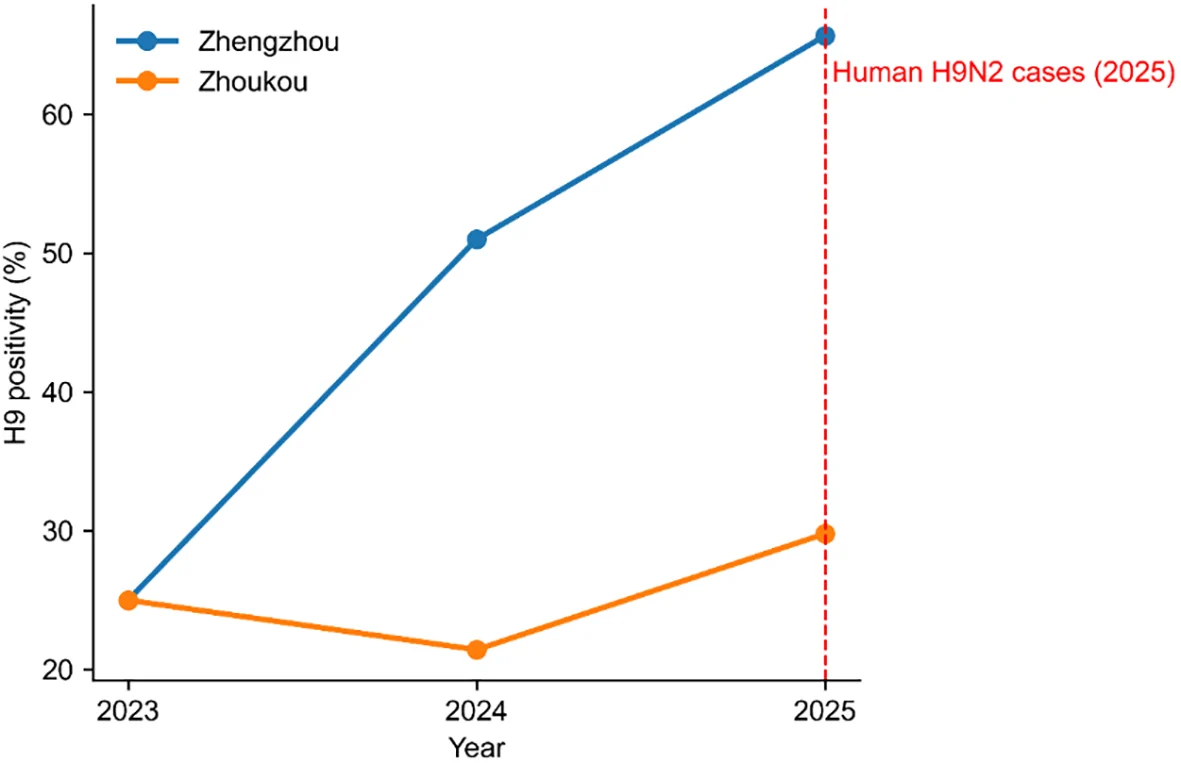
Temporal trends of H9N2 positivity in case cities.

Five human AIV infections were identified during the surveillance period, including H5N6 and H3N8 in 2022 and three H9N2 cases in 2025 (**Table 2**). Most patients were children (4/5), and all reported exposure to live poultry. Comparison of environmental H9N2 positivity before and after the identification of human cases revealed an increasing trend in both Zhoukou (Case 4) and Zhengzhou (Case 5) during 2023–2025, with peak detection levels observed in 2025, when human H9N2 infections were also identified (**Figure 2**). Serological results from occupationally exposed populations further identified H3N8-reactive and H9N2-seropositive samples across multiple surveillance years (**Table 3**). H3N8-reactive samples increased after 2022, with the highest number detected in 2023–2024, whereas H9N2-seropositive samples were continuously detected during the surveillance period.

**Table 2:** Characteristics of human AIV infections in Henan Province, 2020–2025.

Case	Date	AIV subtype	Age (years)	Sex	Occupation	Underlying diseases	City of origin	Exposure to live poultry (7–14 days)	Human sample	Environmental sample	Virus isolation	Symptoms	Final outcome
1	22-Mar	H5N6	28	Male	Poultry worker	No	Puyang City	Yes	Positive	Positive	Not obtained	Severe pneumonia	Death
2	22-Apr	H3N8	4	Female	Child	No	Zhumadian City	Yes	Positive	Positive	Human & environment isolates obtained	Upper respiratory infection (URI)	Recovery
3	25-Mar	H9N2 and A(H1N1)pdm09 co-infection	7	Female	Child	No	Shangqiu City	Yes	Positive	Negative	Human isolate obtained	Upper respiratory infection (URI)	Recovery
4	25-May	H9N2	52	Female	Farmer	Yes (bronchiectasis and hemoptysis)	Zhoukou City	Yes	Positive	Negative	Not obtained	Hemoptysis	Recovery
5	25-Oct	H9N2	3	Female	Child	No	Zhengzhou City	Yes	Positive	Positive	Human & environment isolates obtained	Upper respiratory infection (URI)	Recovery

**Table 3:** Serological surveillance results among occupationally exposed populations during 2020–2025.

Surveillance year	No. of samples	H3N8 HI ≥ 10	H3N8 MN ≥ 10	H9N2 HI ≥ 80	H9N2 MN ≥ 80
2020–2021	471	ND	ND	1	0
2021–2022	486	1	1	7	3
2022–2023	484	8	7	6	3
2023–2024	847	27	24	22	6
2024–2025	842	2	0	11	2

ND, not determined. H3N8 assays were not performed during 2020–2021. H3N8 results were interpreted as serological reactivity due to the lack of established serological criteria. H9N2 seropositivity was defined as both HI and MN titers ≥ 80.

### 3.3 Molecular epidemiological characteristics of H3N8 viruses

**Figure 3:**
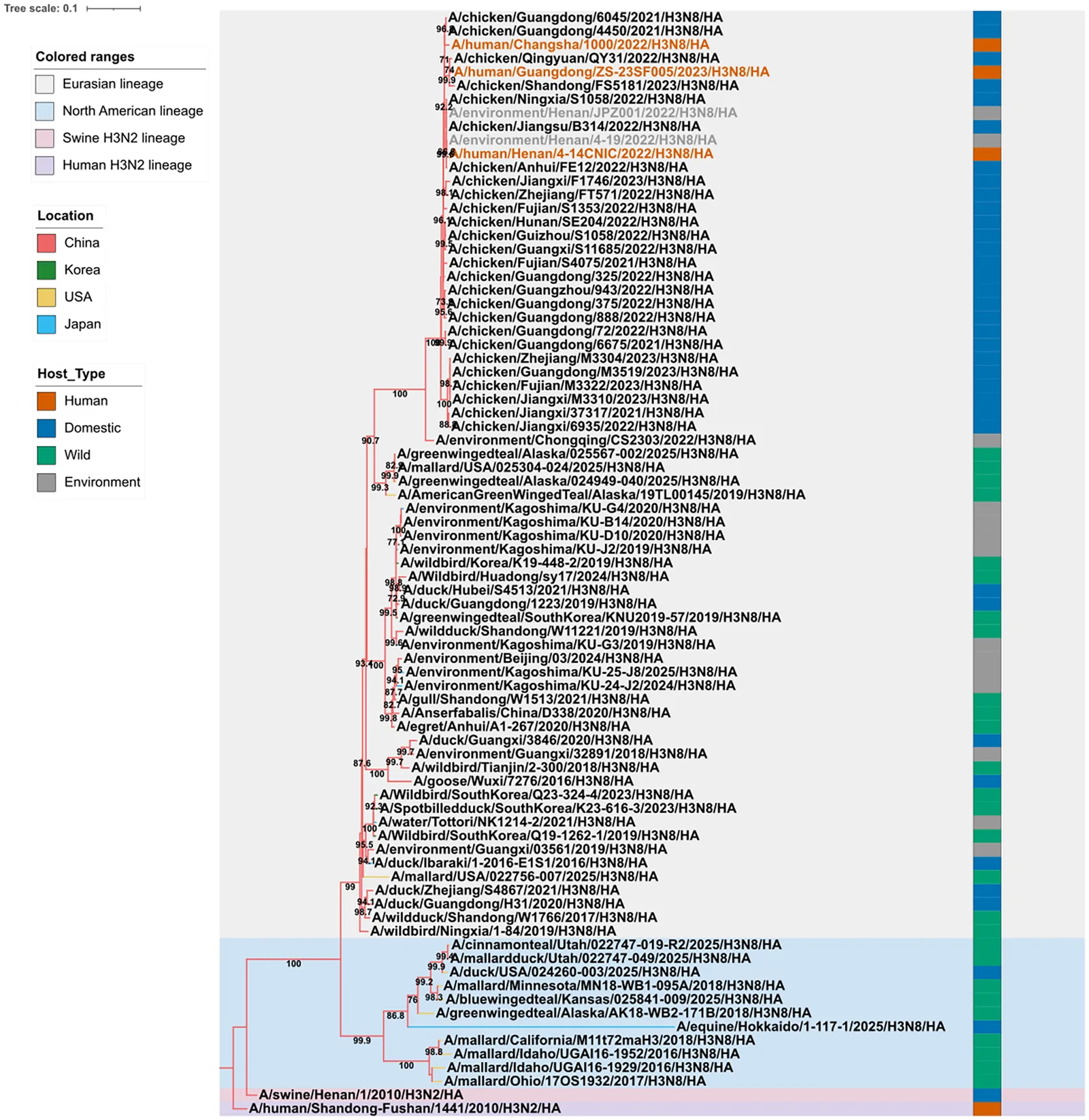
Maximum likelihood phylogenetic tree of the HA gene of H3N8 viruses.

Three H3N8 viruses were identified, including one human-derived virus, one exposure-related environmental virus, and one environmental virus collected approximately one month before the identification of the human case. These viruses showed high nucleotide similarity across all eight gene segments (99.59–100%). Pairwise SNP analysis showed that the human-derived virus differed from the exposure-associated environmental virus and the environmental surveillance virus by 17 and 23 nucleotide substitutions across the eight gene segments, respectively (**Supplementary Table 4**). Phylogenetic analysis showed that the three viruses clustered within a single phylogenetic clade with strong bootstrap support and were grouped with avian-origin H3N8 viruses reported in Henan, Jiangsu, Anhui, Jiangxi, and Fujian provinces in 2022 (**Table 4**; **Figure 3**). Genetic analysis revealed that the HA gene belonged to the Eurasian lineage, whereas the NA gene was of North American lineage origin. All internal genes were genetically related to co-circulating H9N2 viruses detected in Henan and other regions of China (**Figure 3**; **Supplementary Figures 2**–**8**). Comparative analysis of key amino acid substitutions showed that the environmental H3N8 viruses retained receptor-binding patterns typically observed in avian influenza viruses and did not harbor additional mammalian-adaptive markers compared with the human-derived virus (**Supplementary Table 3**).

**Table 4:** Nucleotide sequence identity (%) among H3N8 viruses from different sources.

Gene	Human vs environmental	Human vs surveillance	Environmental vs surveillance
PB2	99.82	99.91	99.91
PB1	99.87	99.78	99.74
PA	100	99.91	99.91
HA	99.94	99.82	99.88
NP	99.87	99.73	99.87
NA	99.86	99.93	99.79
MP	99.9	99.7	99.59
NS	99.76	99.88	99.64

Human: virus isolated from the confirmed human case; Environmental: virus isolated from environmental samples linked to the exposure event of the human case; Surveillance: environmental virus isolated through routine avian influenza surveillance conducted approximately one month before the onset of the human infection in different cities within Henan Province.

### 3.4 Genetic diversity and evolutionary characteristics of H9N2 viruses

**Figure 4:**
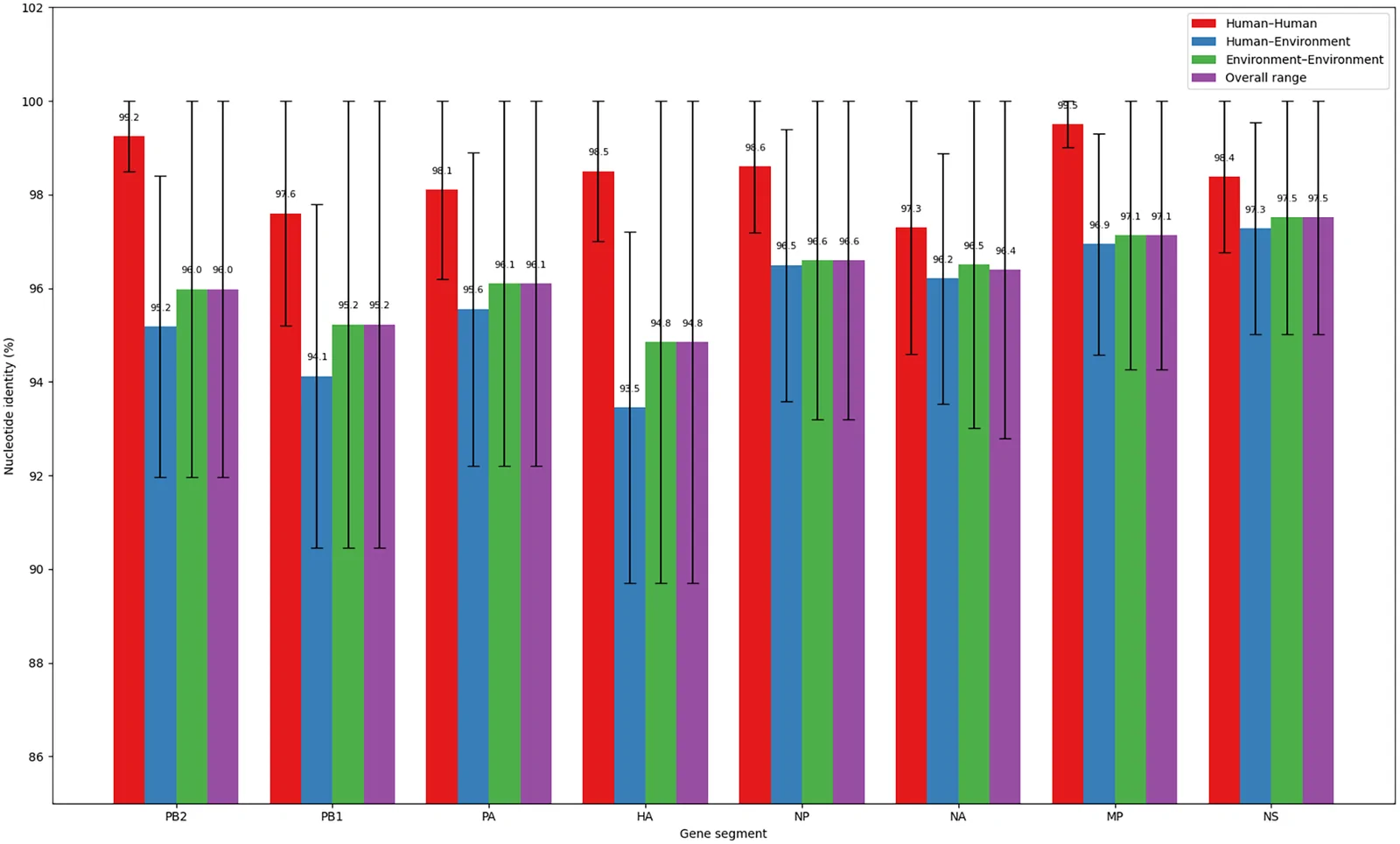
Pairwise nucleotide identity comparisons of H9N2 viruses. Sequence identities were compared among human-human, human-environment, and environment-environment groups across all gene segments.

**Figure 5:**
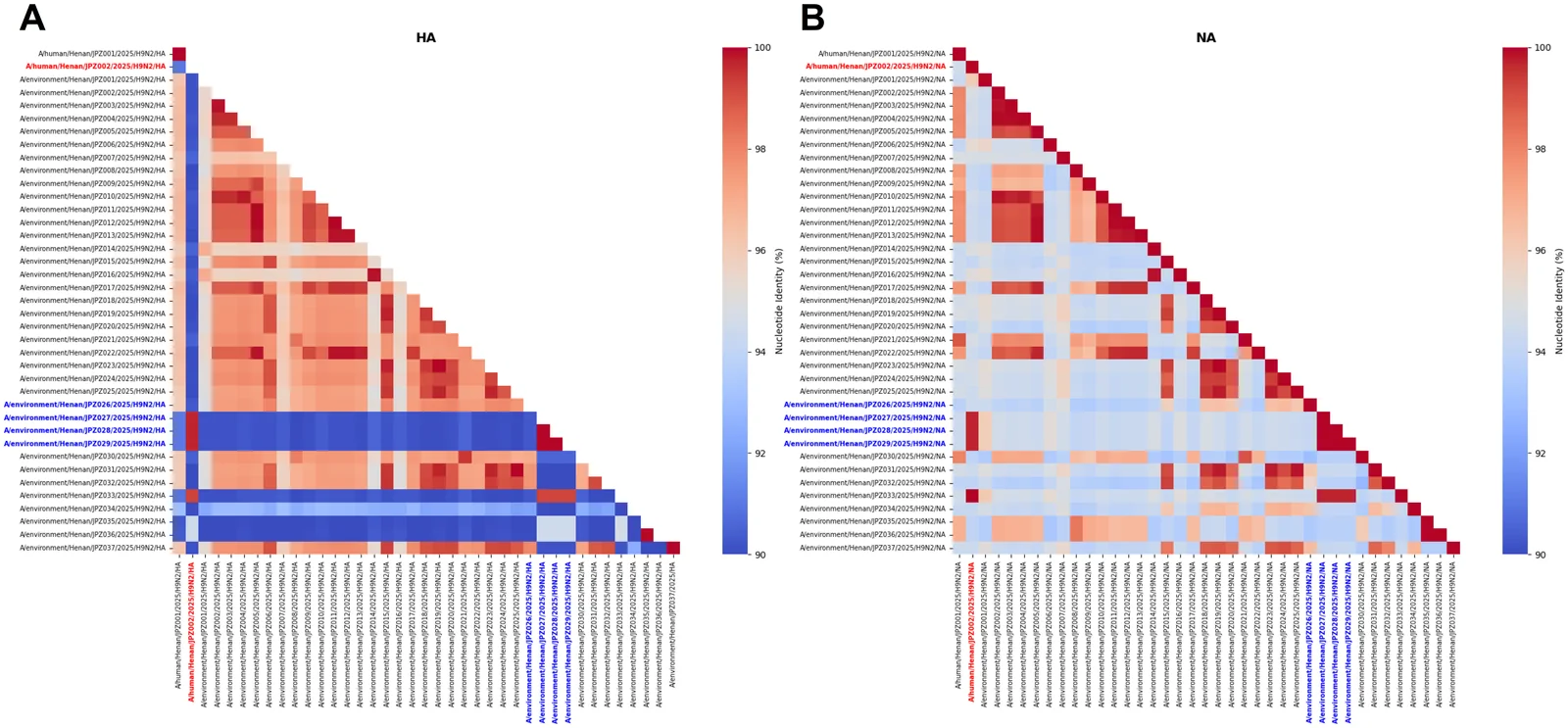
Heatmap of pairwise nucleotide identities of HA and NA genes among H9N2 viruses. **(A)** HA genes; **(B)** NA genes. Red indicates the case isolate, and blue indicates environmental isolates associated with the exposure of the case

**Figure 6:**
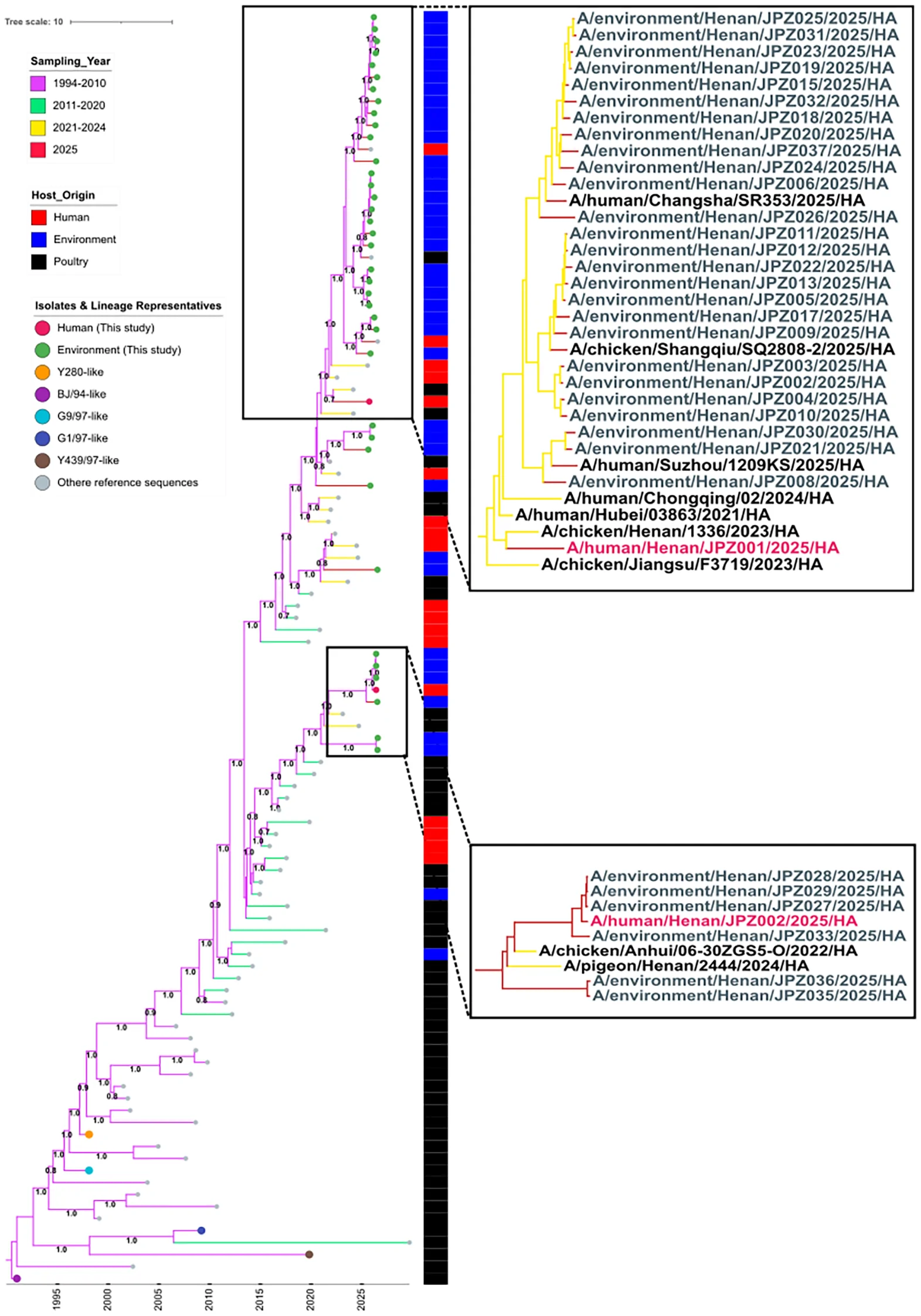
Time-scaled maximum clade credibility (MCC) tree of the HA gene of H9N2 avian influenza viruses in Henan Province.

A total of 39 H9N2 viruses were obtained in 2025, including 2 human-derived and 37 environmental isolates. Environmental viruses displayed broader genetic diversity than human-derived viruses, with the HA gene showing the greatest variability, whereas the M and NS genes were relatively conserved (**Figure 4**). Pairwise genomic comparison revealed high nucleotide similarity between the Case 5 human isolate and several environmental H9N2 viruses detected in exposure-related environments and a neighboring prefecture (**Figure 5**; **Supplementary Figures 9**–**11**). SNP analysis further demonstrated limited genomic divergence, with only 35 nucleotide substitutions identified across the eight gene segments between the human isolate and JPZ027–JPZ029 (**Supplementary Table 5**). Phylogenetic analysis showed that human-derived viruses were distributed within the diversity of environmental H9N2 lineages and clustered with genetically related environmental viruses with strong bootstrap support (> 70%) (**Figure 6**; **Supplementary Figures 12**–**18**). All HA and NA genes of the analyzed viruses belonged to the Y280-like lineage and were classified as the G57 genotype. The PB1, PA, NP, and NS segments were derived from the F/98-like lineage, whereas PB2 and M segments originated from the G1/97-like lineage. The tMRCA of the HA and NA genes was estimated to be around 2017 (95% HPD: 2015–2019), with mean evolutionary rates of 4.92 × 10^−3^ and 5.31 × 10^−3^ substitutions/site/year, respectively. These genomic similarities indicate close genetic relatedness between human and environmental isolates, yet cannot verify direct transmission chains.

### 3.5 Genetic characteristics and key amino acid substitutions in H9N2 viruses

**Figure 7:**
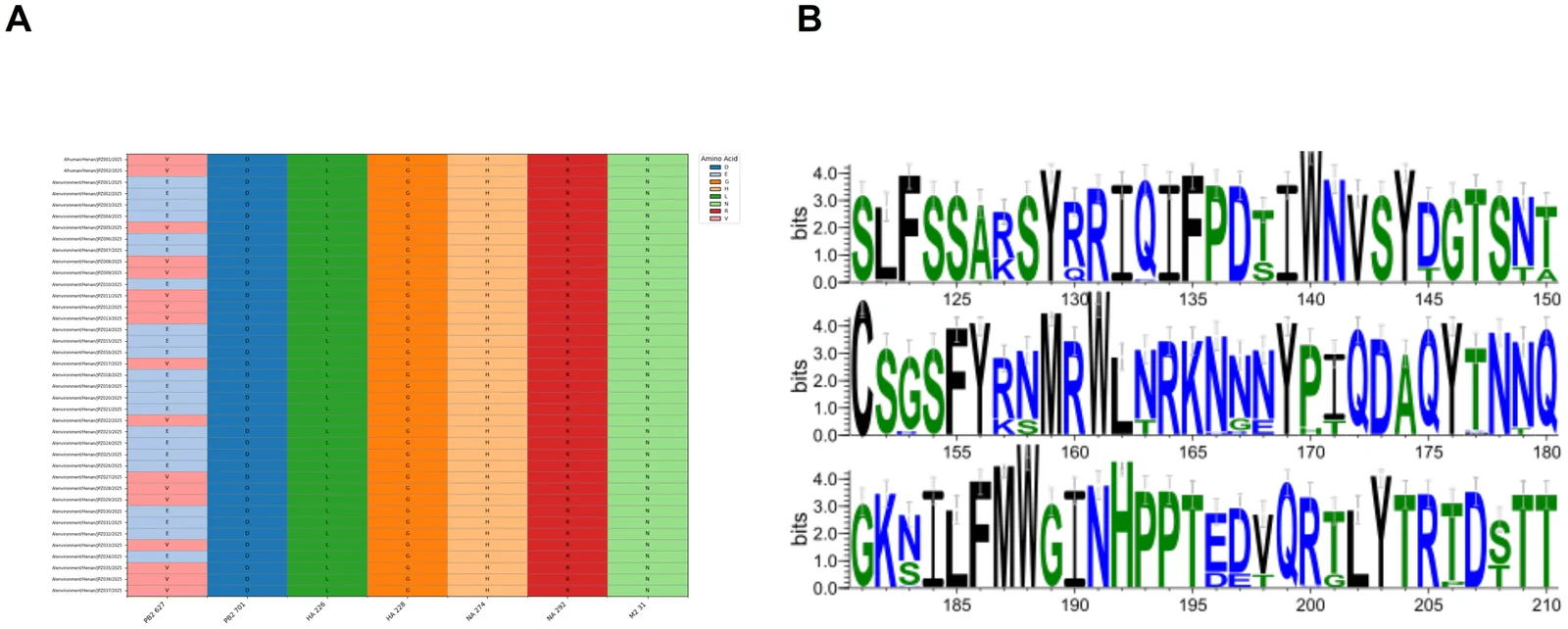
Genetic characteristics and key amino acid substitutions of H9N2 avian influenza viruses (AIVs) in Henan Province. **(A)** Heatmap of key amino acid mutation sites. **(B)** Web Logo representation of amino acid frequencies at key sites.

Key molecular markers associated with mammalian adaptation, receptor binding, and antiviral resistance were analyzed among the 39 H9N2 viruses (**Figure 7A**). The PB2-E627V substitution, which has been reported to affect polymerase activity and replication efficiency in mammalian models, was detected in 43.59% (17/39) of viruses. In contrast, classical mammalian adaptation markers, including PB2-D701N, receptor-binding associated substitutions such as HA-G228S, and neuraminidase inhibitor resistance markers NA-H274Y and NA-R292K, were not identified. A complete profile of amino acid substitutions across all viral proteins is provided in **Supplementary Table 6**.

Analysis of the HA receptor-binding domain (aa 121–210) revealed conserved residues at positions 147, 164, and 207, whereas variability was observed at multiple sites (**Figure 7B**; **Table 5**). Dominant amino acid patterns were identified at several variable positions, including D145 and N168, each detected in 82.05% (32/39) of viruses. Coordinated variation was observed at positions 168, 197, and 201 in a subset of viruses, with the 168E/197E/201G combination detected in both environmental and human-derived isolates, suggesting that human-associated viruses shared molecular features with H9N2 viruses circulating in poultry-associated environments.

**Table 5:** Amino acid substitutions of H9N2 isolates at key positions (n = 39).

Position	Reference F/98	Reference G1	Amino acid substitutions (% of isolates, n=39)
145	S	T	D (82.05), T (17.95)
147	T	I	T (100.00)
149	K	R	N (82.05), T (17.95)
153	D	G	G (94.87), D (5.13)
164	Q	Q	R (100.00)
167	N	G	N (79.49), G (15.38), D (5.13)
168	A	F	N (82.05), E (17.95)
196	D	Y	E (79.49), D (20.51)
197	T	T	D (82.05), E (17.95)
200	T	T	R (97.44), Q (2.56)
201	N	N	T (82.05), G (17.95)
207	D	D	D (100.00)

## 4 Discussion

In this six-year longitudinal surveillance study, we characterized the circulation of multiple AIV subtypes across poultry-associated environments and investigated their potential zoonotic implications by integrating environmental, serological, and genomic evidence. Our findings highlight the continued circulation of diverse AIVs at the human-animal interface in central China, where live poultry production and trading were associated with sustained environmental AIV detection and potential zoonotic exposure (Cui et al., 2021; Hu et al., 2025). Consistent with previous reports, AIVs were frequently detected in LPMs despite existing control measures, highlighting the continued importance of these settings as potential sources of human exposure (Bo et al., 2025). Notably, most human cases occurred in children, suggesting that exposure may occur beyond traditional occupational settings (Bo et al., 2026). Within LPMs, we further observed that processing-related environments, including defeathering machine swabs and poultry washing wastewater, exhibited higher AIV detection rates and relatively higher co-detection frequencies. Such environments may provide ecological conditions favorable for viral persistence and reassortment (Bi et al., 2020; Dharmayanti et al., 2025). Previous investigations of LPMs have indicated that variations in market environments and operational practices contribute to differences in AIV contamination levels, suggesting that improving hygiene management and biosecurity conditions may help limit viral persistence in these settings (Islam et al., 2023). Together, these findings highlight that environmental surveillance combined with targeted biosecurity interventions is essential for early detection, risk reduction, and prevention of zoonotic influenza under a One Health framework.

Multiple avian influenza subtypes, including H3, H4, H5, H7, and H9, were detected during the study period, with environmental positivity increasing markedly during 2024–2025 (Gao, 2014). H9N2 viruses have remained enzootic in poultry populations despite extensive vaccination programs, suggesting that current control measures may reduce disease severity but may not completely prevent viral circulation or environmental contamination. Consistent with previous studies, H9N2 viruses continue to evolve under multiple selective pressures, including immune selection associated with vaccination (Savill et al., 2006; Salaheldin et al., 2026). Therefore, vaccination should be complemented by continuous veterinary surveillance, improved biosecurity, and risk-based interventions to monitor viral evolution and reduce zoonotic risks (Thomas et al., 2022). In our surveillance, H9N2 viruses remained the predominant subtype and consistently showed high detection rates, coinciding with three human H9N2 infections during the same period. All H9N2 viruses identified in this study belonged to the G57 lineage, which has circulated in China for more than a decade (Bi et al., 2020; Yang et al., 2025b). The detection of this dominant lineage in environmental samples, together with the identification of human infections during the same period, further indicates continued circulation of G57-like H9N2 viruses at the animal-environment-human interface. One of the three H9N2 cases was co-infected with seasonal influenza A(H1N1)pdm09 virus. Although no further characterization of the seasonal influenza virus was performed in this study, the detection of co-infection highlights the importance of comprehensive influenza surveillance to identify concurrent infections and improve understanding of the co-circulation of avian and seasonal influenza viruses. For Case 4, although H9N2 viral sequences were identified from the respiratory specimen, no H9N2-positive environmental samples were detected during the traceback investigation of investigated environments associated with the reported exposure history. The delayed sampling after symptom onset and temporal variation in environmental virus circulation may partly explain the lack of environmental confirmation. Therefore, the absence of environmental confirmation does not exclude potential exposure to H9N2 viruses. Beyond environmental and case-based investigations, serological surveillance provides an additional approach to assess population-level exposure to avian influenza viruses (De Marco et al., 2025; Power et al., 2026). In this study, H9N2-seropositive individuals were identified among occupationally exposed populations across multiple years, providing complementary evidence of previous H9N2 virus exposure. Together with environmental surveillance and human case findings, these serological results support the continued presence of H9N2 exposure risk at the animal-environment-human interface. However, comparisons of H9N2 seropositivity across years should be interpreted cautiously because different reference viruses were used during different surveillance periods to better represent the predominant circulating strains. Antigenic differences among reference strains may influence serological reactivity and limit direct comparison of seropositivity across years. Therefore, these findings mainly indicate sustained H9N2 exposure among poultry-exposed populations rather than precise temporal changes in seroprevalence. In addition, H3N8-reactive samples increased during 2022–2024, suggesting possible exposure to H3 or antigenically related influenza viruses among poultry-exposed populations. However, standardized serological criteria for H3N8 viruses are still unavailable, and potential cross-reactive antibodies induced by other influenza viruses cannot be excluded.

Notably, in April 2022, the first human H3N8 virus infection (case 2) was reported in Zhumadian, Henan Province (Cheng et al., 2022). Epidemiological investigation indicated that the associated live poultry market was supplied by poultry from multiple provinces, including Jiangsu and Anhui. In this study, H3N8 detection remained rare, with only a single environmental isolate obtained approximately one month before the case (Yang et al., 2022; Yang et al., 2025a). Phylogenetic analysis showed that the human strain, the exposure-related environmental virus, and the pre-case environmental isolate clustered within the same clade and were closely related to chicken-origin viruses from Henan, Jiangsu, and Anhui, suggesting that genetically related H3N8 viruses were circulating among poultry-associated environments in these regions. These phylogenetic findings contribute to our understanding of the molecular epidemiology and genetic relationships of viruses from different hosts and regions and provide insights into viral evolution associated with zoonotic influenza events. These viruses also carried internal gene segments derived from co-circulating H9N2 strains, indicating that reassortment involving H9N2-like internal genes may have contributed to their genetic composition (Hao et al., 2025; Yang et al., 2025). Genetic typing revealed a characteristic mixed-lineage genome architecture for these H3N8 viruses: Eurasian-lineage HA paired with North American-lineage NA, combined with six internal gene segments originating from locally prevalent H9N2 viruses. This specific constellation matches the well-defined triple reassortant H3N8 genotype repeatedly identified from human spillover cases nationwide in previous studies (Yang et al., 2023; Zhao et al., 2025). Eurasian and North American avian influenza virus reservoirs are geographically segregated by transcontinental migratory routes, making inter-lineage gene reassortment a relatively rare natural event. The detection of such strains in central China confirms that migratory wild birds act as natural vectors to introduce North American-origin N8 gene segments into Eurasian viral ecosystems. Combined with H9N2-derived internal genes widespread in domestic poultry, our data support a two-step reassortment model: initial reassortment between Eurasian H3 viruses and N8-carrying strains of North American ancestry, followed by secondary reassortment with enzootic H9N2 viruses in domestic poultry environments. Successive multi-source reassortment reshapes viral genomic composition and may alter biological phenotypes, creating novel zoonotic influenza variants with spillover capacity. This integrated environmental, epidemiological and genomic evidence provides important support for the circulation of H3N8 viruses in poultry-associated environments around the time of human infection and highlights the value of a One Health approach for investigating zoonotic influenza events. Importantly, no further H3N8 viruses were detected after the case, which is consistent with a sporadic human infection event rather than evidence of sustained human-to-human transmission. The genetic similarity between human and environmental isolates suggests a potential epidemiological association with local poultry-associated environments and is consistent with possible exposure to genetically related viruses (Yang et al., 2022). Notably, Case 5 also showed close phylogenetic relatedness to a strain from a neighboring prefecture, suggesting the circulation of genetically related viruses across geographically adjacent areas. Overall, these findings indicate that detection of genetically related avian viruses in LPMs and across geographically adjacent regions was associated with potential human exposure risk (Bao et al., 2013; Duan et al., 2023). Strengthening environmental surveillance in these settings may improve early detection of changes in viral activity (Blagodatski et al., 2021; Dharmayanti et al., 2025).

Emerging influenza viruses capable of replicating in humans often acquire key amino acid substitutions to achieve host adaptation. Some of these adaptive mutations also enhance the virus’s ability to spread among humans (Liu et al., 2022). For example, human H7N9 infections in 2013 were associated with receptor-binding substitutions (HA-Q226L and G228S), and mammalian adaptation markers (PB2-E627K and D701N) (Li and Chen, 2021). Previous studies have shown that human-derived H3N8 virus strains carried the HA-G228S and PB2-E627K substitutions (Cui et al., 2023; Sun et al., 2023; Zhu et al., 2023). However, these substitutions were absent in the H3N8 viruses analyzed in this study, including exposure-associated environmental strains linked to the human case and routine surveillance samples collected one month earlier, indicating that additional viral and epidemiological factors may contribute to zoonotic infection. In comparison, the H9N2 viruses analyzed in this study exhibited several adaptation-associated characteristics. All H9N2 viruses carried the HA-Q226L substitution, and nearly half of them harbored the PB2-E627V mutation. Previous studies have demonstrated that the PB2-E627V substitution enhances replication and virulence of diverse avian influenza viruses in mice (Song et al., 2025; Zhang et al., 2025). In addition, all viruses carried the M2-S31N substitution associated with adamantane resistance, whereas neuraminidase inhibitor resistance markers NA-H274Y and NA-R292K were absent. Despite these differences in molecular characteristics, human infections were observed for both H3N8 and H9N2 viruses. This finding suggests that acquisition of the complete set of classical mammalian adaptation markers may not be a prerequisite for zoonotic infection. Instead, zoonotic infection risk may be influenced by a combination of viral genetic characteristics and the intensity of exposure at the human-animal interface. Overall, these observations indicate that avian influenza viruses with limited adaptive changes may still pose a potential zoonotic risk under conditions of sustained exposure (Li et al., 2018; Wang et al., 2025).

Several limitations of this study should be noted. First, the number of confirmed human avian influenza cases identified during the surveillance period was small, and despite a considerable number of nucleic acid-positive environmental samples, only a subset yielded complete viral genomes, restricting further epidemiological and virological analyses as well as comprehensive investigations of viral evolution and reassortment. Second, serological surveillance relied on convenience sampling of occupationally exposed populations rather than random sampling, which may introduce selection bias and limit population-level extrapolation. Third, human avian influenza case detection depended on passive surveillance, and mild or asymptomatic infections may therefore have been missed. Fourth, surveillance sites and sampling intensity varied during the study period, which may have introduced sampling bias. Finally, although genetic relationships were observed between human-derived and environmental viruses, systematic poultry host sampling and viral phenotypic characterization were not performed, limiting our ability to determine transmission direction and further evaluate the biological properties of the detected viruses.

In conclusion, LPMs in Henan Province remain key settings for avian influenza virus circulation and human exposure (Bo et al., 2025). Although control measures have been implemented, AIVs continued to be detected in poultry-associated environments, highlighting the need for sustained surveillance to characterize viral circulation patterns and assess potential risks at the human-animal interface (Zhang et al., 2025). Routine environmental surveillance, particularly in high-contact areas such as slaughtering and processing sites, should be integrated with animal and human influenza surveillance to facilitate early detection of emerging viruses and timely risk assessment (Badra et al., 2025; Chou et al., 2025). Monitoring wild birds and other potential reservoirs is also important because these populations may contribute to the introduction and diversification of influenza viruses within poultry-associated ecosystems (Pardini et al., 2026). This six-year integrated surveillance study confirms that multidimensional monitoring covering animal populations, environmental matrices, migratory wildlife reservoirs, and human serological and clinical indicators is essential for systematically understanding AIV ecological dynamics, tracking viral genetic variation, and achieving precise zoonotic risk assessment. Strengthening LPM biosecurity management, standardized environmental disinfection, cross-regional poultry trade supervision, wild bird dynamic monitoring, and occupational population protection will further improve the regional One Health prevention and control system. Overall, our findings strongly support that coordinated cross-sector surveillance and multi-level intervention strategies are indispensable for long-term zoonotic influenza prevention and emerging viral threat early warning in central China (Zheng et al., 2019; Pardini et al., 2026).

## Data Availability

The datasets presented in this study can be found in online repositories. The names of the repository/repositories and accession number(s) can be found in the article/**Supplementary Material**.
