# Supplementary material for: Environmental circulation of multiple avian influenza subtypes and potential association with human infections in central China
Source: Front Cell Infect Microbiol. 2026 Sep 10;16:1911586. doi: 10.3389/fcimb.2026.1911586 (PMC13600820; doi:10.3389/fcimb.2026.1911586)
Supplement: Supplementary file 1 [file SupplementaryFile1.docx]

**Supplementary Figure 1 Environmental surveillance of avian influenza viruses (AIVs) in Henan Province, 2020-2025**. (A) Positivity rates of AIV by sampling source. **(B)** Positivity rates by sample type. LPMs: live poultry markets; BPFs: backyard poultry flocks; CPFs: commercial poultry farms; PSPs: poultry slaughtering and processing plants; WBHs: wild bird habitats. PDW: poultry drinking water; F: feces; CSS: cage surface swabs; CBS: chopping board swabs; PWW: poultry washing wastewater; DMS: defeathering machine swabs; Fe: feathers; ESS: eggshell swabs.

**Supplementary Figure 2 Maximum likelihood phylogenetic tree of the NA gene of H3N8 viruses.**

**Supplementary Figure 3 Maximum likelihood phylogenetic tree of the PB2 gene of H3N8 viruses.**

**Supplementary Figure 4 Maximum likelihood phylogenetic tree of the PB1 gene of H3N8 viruses.**

**Supplementary Figure 5 Maximum likelihood phylogenetic tree of the PA gene of H3N8 viruses.**

**Supplementary Figure 6 Maximum likelihood phylogenetic tree of the NP gene of H3N8 viruses.**

**Supplementary** **Figure 7 Maximum likelihood phylogenetic tree of the MP gene of H3N8 viruses.**

**Supplementary Figure 8 Maximum likelihood phylogenetic tree of the NS gene of H3N8 viruses.**

**Supplementary** **Figure 9** **Heatmap of pairwise nucleotide identities of PB2 (A) and PB1 (B) genes among H9N2 viruses.**

**Supplementary Figure 10** **Heatmap of pairwise nucleotide identities of PA (A) and NP (B) genes among H9N2 viruses.**

**Supplementary Figure 11** **Heatmap of pairwise nucleotide identities of MP (A) and NS (B) genes among H9N2 viruses.**

**Supplementary Figure 12** **Time-scaled maximum clade credibility (MCC) tree of the NA gene of H9N2 avian influenza viruses in Henan Province.**

**Supplementary Figure 13 Maximum likelihood phylogenetic tree of the PB2 gene of H9N2 viruses.**

**Supplementary Figure 14 Maximum likelihood phylogenetic tree of the PB1 gene of H9N2 viruses.**

**Supplementary** **Figure 15** Maximum likelihood phylogenetic tree of the PA gene of H9N2 viruses.

**Supplementary Figure 16 Maximum likelihood phylogenetic tree of the NP gene of H9N2 viruses.**

**Supplementary Figure 17 Maximum likelihood phylogenetic tree of the MP gene of H9N2 viruses.**

**Supplementary Figure 18 Maximum likelihood phylogenetic tree of the NS gene of H9N2 viruses.**
