# Supplementary material for: Environmental circulation of multiple avian influenza subtypes and potential association with human infections in central China
Source: Front Cell Infect Microbiol. 2026 Sep 10;16:1911586. doi: 10.3389/fcimb.2026.1911586 (PMC13600820; doi:10.3389/fcimb.2026.1911586)
Supplement: Supplementary file 2 [file Table1.docx]

**Supplementary Table 1 Distribution of environmental surveillance sites across prefecture-level cities in Henan Province, China, 2020-2025**

| **City** | **Surveillance years** | **No. of samples collected** | **LPMs** | **BPFs** | **CPFs** | **PSPs** | **WBHs** |
| --- | --- | --- | --- | --- | --- | --- | --- |
| Anyang | 2020 | 195 | 1 | 2 | 1 | 0 | 0 |
| Hebi | 2023–2025 | 469 | 1 | 1 | 1 | 1 | 0 |
| Jiyuan | 2020–2025 | 569 | 1 | 0 | 1 | 1 | 0 |
| Jiaozuo | 2020–2021, 2023–2025 | 535 | 4 | 1 | 4 | 0 | 0 |
| Luoyang | 2020–2025 | 1,497 | 2 | 1 | 2 | 1 | 0 |
| Luohe | 2020–2025 | 574 | 1 | 0 | 1 | 1 | 0 |
| Nanyang | 2020 | 162 | 1 | 1 | 1 | 1 | 0 |
| Pingdingshan | 2020 | 105 | 1 | 0 | 1 | 0 | 0 |
| Puyang | 2020, 2023–2025 | 551 | 2 | 0 | 3 | 1 | 0 |
| Sanmenxia | 2020–2025 | 519 | 1 | 0 | 1 | 0 | 0 |
| Shangqiu | 2020 | 219 | 1 | 1 | 1 | 0 | 0 |
| Xinxiang | 2020–2021 | 136 | 4 | 0 | 0 | 0 | 0 |
| Xinyang | 2023–2025 | 428 | 2 | 1 | 1 | 0 | 0 |
| Xuchang | 2020–2025 | 538 | 1 | 1 | 1 | 0 | 0 |
| Zhengzhou | 2020–2021, 2023–2025 | 455 | 3 | 1 | 1 | 1 | 1 |
| Zhoukou | 2020–2025 | 534 | 2 | 0 | 1 | 0 | 0 |
| Zhumadian | 2020, 2023–2025 | 470 | 2 | 1 | 1 | 1 | 0 |
| Total | – | 7,956 | 30 | 11 | 22 | 8 | 1 |

LPMs, live poultry markets; BPFs, backyard poultry flocks; CPFs, commercial poultry farms; PSPs, poultry slaughtering and processing plants; WBHs, wild bird habitats.

**Supplementary Table 2 Characteristics of influenza A viruses included in this study**

| **Viruses** | **Sampling Date** | **Sample type** | **Host** | **Location** | **Accession ID** |
| --- | --- | --- | --- | --- | --- |
| A/environment/Henan/JPZ001/2022/H3N8 | 2022-03-16 | DMS | Environment | Xuchang | EPI_ISL_20435147 |
| A/human/Henan/JPZ001/2025/H9N2 | 2025-02-11 | TS | Human | Shangqiu | EPI_ISL_20451356 |
| A/human/Henan/JPZ002/2025/H9N2 | 2025-10-16 | TS | Human | Zhengzhou | EPI_ISL_20435109 |
| A/environment/Henan/JPZ001/2025/H9N2 | 2025-01-08 | PWW | Environment | Jiaozuo | EPI_ISL_20435110 |
| A/environment/Henan/JPZ002/2025/H9N2 | 2025-01-17 | CSS | Environment | Zhengzhou | EPI_ISL_20435111 |
| A/environment/Henan/JPZ003/2025/H9N2 | 2025-02-11 | CBS | Environment | Zhengzhou | EPI_ISL_20435112 |
| A/environment/Henan/JPZ004/2025/H9N2 | 2025-02-18 | CBS | Environment | Jiaozuo | EPI_ISL_20435113 |
| A/environment/Henan/JPZ005/2025/H9N2 | 2025-03-12 | CBS | Environment | Luoyang | EPI_ISL_20435114 |
| A/environment/Henan/JPZ006/2025/H9N2 | 2025-03-19 | CSS | Environment | Xinyang | EPI_ISL_20435117 |
| A/environment/Henan/JPZ007/2025/H9N2 | 2025-03-20 | CSS | Environment | Sanmenxia | EPI_ISL_20435118 |
| A/environment/Henan/JPZ008/2025/H9N2 | 2025-03-24 | PWW | Environment | Xinyang | EPI_ISL_20435119 |
| A/environment/Henan/JPZ009/2025/H9N2 | 2025-04-15 | Fe | Environment | Zhoukou | EPI_ISL_20435115 |
| A/environment/Henan/JPZ010/2025/H9N2 | 2025-04-17 | CSS | Environment | Zhengzhou | EPI_ISL_20435116 |
| A/environment/Henan/JPZ011/2025/H9N2 | 2025-04-17 | PDW | Environment | Luoyang | EPI_ISL_20435120 |
| A/environment/Henan/JPZ012/2025/H9N2 | 2025-04-30 | DMS | Environment | Luohe | EPI_ISL_20435121 |
| A/environment/Henan/JPZ013/2025/H9N2 | 2025-05-08 | CSS | Environment | Jiaozuo | EPI_ISL_20435122 |
| A/environment/Henan/JPZ014/2025/H9N2 | 2025-05-12 | CSS | Environment | Zhengzhou | EPI_ISL_20435123 |
| A/environment/Henan/JPZ015/2025/H9N2 | 2025-05-27 | DMS | Environment | Luohe | EPI_ISL_20435124 |
| A/environment/Henan/JPZ016/2025/H9N2 | 2025-06-09 | PDW | Environment | Zhengzhou | EPI_ISL_20435125 |
| A/environment/Henan/JPZ017/2025/H9N2 | 2025-06-16 | Fe | Environment | Zhoukou | EPI_ISL_20435126 |
| A/environment/Henan/JPZ018/2025/H9N2 | 2025-06-27 | F | Environment | Luohe | EPI_ISL_20435127 |
| A/environment/Henan/JPZ019/2025/H9N2 | 2025-07-24 | DMS | Environment | Luohe | EPI_ISL_20435128 |
| A/environment/Henan/JPZ020/2025/H9N2 | 2025-08-04 | CBS | Environment | Zhumadian | EPI_ISL_20435129 |
| A/environment/Henan/JPZ021/2025/H9N2 | 2025-08-11 | DMS | Environment | Xuchang | EPI_ISL_20435130 |
| A/environment/Henan/JPZ022/2025/H9N2 | 2025-08-14 | PDW | Environment | Zhengzhou | EPI_ISL_20435131 |
| A/environment/Henan/JPZ023/2025/H9N2 | 2025-08-27 | CBS | Environment | Luohe | EPI_ISL_20435132 |
| A/environment/Henan/JPZ024/2025/H9N2 | 2025-09-18 | DMS | Environment | Zhoukou | EPI_ISL_20435133 |
| A/environment/Henan/JPZ025/2025/H9N2 | 2025-09-29 | DMS | Environment | Luohe | EPI_ISL_20435134 |
| A/environment/Henan/JPZ026/2025/H9N2 | 2025-10-16 | CSS | Environment | Zhengzhou | EPI_ISL_20435135 |
| A/environment/Henan/JPZ027/2025/H9N2 | 2025-10-16 | CSS | Environment | Zhengzhou | EPI_ISL_20435136 |
| A/environment/Henan/JPZ028/2025/H9N2 | 2025-10-16 | CSS | Environment | Zhengzhou | EPI_ISL_20435137 |
| A/environment/Henan/JPZ029/2025/H9N2 | 2025-10-16 | PDW | Environment | Zhengzhou | EPI_ISL_20435138 |
| A/environment/Henan/JPZ030/2025/H9N2 | 2025-10-23 | CBS | Environment | Luoyang | EPI_ISL_20435139 |
| A/environment/Henan/JPZ031/2025/H9N2 | 2025-11-13 | DMS | Environment | Luohe | EPI_ISL_20435140 |
| A/environment/Henan/JPZ032/2025/H9N2 | 2025-11-18 | CSS | Environment | Zhoukou | EPI_ISL_20435141 |
| A/environment/Henan/JPZ033/2025/H9N2 | 2025-11-26 | CBS | Environment | Jiaozuo | EPI_ISL_20435142 |
| A/environment/Henan/JPZ034/2025/H9N2 | 2025-12-02 | CBS | Environment | Luoyang | EPI_ISL_20435143 |
| A/environment/Henan/JPZ035/2025/H9N2 | 2025-12-04 | CSS | Environment | Xinyang | EPI_ISL_20435144 |
| A/environment/Henan/JPZ036/2025/H9N2 | 2025-12-12 | PWW | Environment | Xinyang | EPI_ISL_20435145 |
| A/environment/Henan/JPZ037/2025/H9N2 | 2025-12-30 | PDW | Environment | Luohe | EPI_ISL_20435146 |

PDW: poultry drinking water; F: feces; CSS: cage surface swabs; CBS: chopping board swabs; PWW: poultry washing wastewater; DMS: defeathering machine swabs; Fe: feathers; ESS: eggshell swabs.

**Supplementary Table 3 Key amino acid substitutions and functional markers of H3N8 viruses identified in this study**

| **Gene** | **Position** | **Functional significance** | **A/human/Henan/4-14CNIC/2022/H3N8/HA** | **A/human/Changsha/1000/2022/H3N8/HA** | **A/human/Guangdong/ZS-23SF005/2023/H3N8/HA** | **A/environment/Henan/4-19/2022/H3N8/HA** | **A/environment/Henan/JPZ001/2022/H3N8/HA** |
| --- | --- | --- | --- | --- | --- | --- | --- |
| PB2 | 591 | Mammalian adaptation | Q | Q | Q | Q | Q |
|  | 627 | Mammalian adaptation | K | V | E | E | E |
|  | 701 | Mammalian adaptation | D | D | D | D | D |
| HA | 226 | Receptor binding (avian vs human) | Q | Q | Q | Q | Q |
|  | 228 | Receptor binding | mixed | G | G | G | G |
|  | Cleavage site | Pathogenicity marker | PEKQTR↓GLF | PEKQTR↓GLF | PEKQTR↓GLF | PEKQTR↓GLF | PEKQTR↓GLF |
| NA | 63–65 | NA stalk deletion (poultry adaptation) | Deletion | Deletion | Deletion | Deletion | Deletion |
|  | 274 | Oseltamivir resistance | H | H | H | H | H |
|  | 292 | NA inhibitor resistance | R | R | R | R | R |
| M2 | 31 | Adamantane resistance | N | N | N | N | N |

**Supplementary Table 4 Pairwise nucleotide differences between the human-derived H3N8 virus and environmental H3N8 isolates across the eight gene segments.**

| **Gene segment** | **Human vs exposure-associated environmental** | **Human vs pre-case environmental** | **Exposure-associated vs pre-case environmental** |
| --- | --- | --- | --- |
| PB2 | 5 | 3 | 2 |
| PB1 | 3 | 5 | 6 |
| PA | 0 | 2 | 2 |
| HA | 2 | 4 | 2 |
| NP | 2 | 4 | 2 |
| NA | 2 | 1 | 3 |
| M | 1 | 3 | 4 |
| NS | 2 | 1 | 3 |
| Total SNP differences | 17 | 23 | 24 |

Human: A/Henan/4-14CNIC/2022(H3N8); exposure-associated environmental: A/environment/Henan/4-19/2022(H3N8); pre-case environmental: A/environment/Henan/JPZ001/2022/H3N8.

**Supplementary Table 5 Pairwise nucleotide differences between the human-derived H9N2 virus and environmental H9N2 viruses across eight gene segments**

| **Gene segment** | **JPZ002 vs JPZ026** | **JPZ002 vs JPZ027** | **JPZ002 vs JPZ028** | **JPZ002 vs JPZ029** |
| --- | --- | --- | --- | --- |
| PB2 | 165 | 2 | 2 | 2 |
| PB1 | 205 | 3 | 3 | 3 |
| PA | 122 | 7 | 7 | 7 |
| HA | 164 | 5 | 5 | 5 |
| NP | 64 | 9 | 9 | 9 |
| NA | 80 | 4 | 4 | 4 |
| MP | 16 | 0 | 0 | 0 |
| NS | 38 | 5 | 5 | 5 |
| Total SNP differences | 854 | 35 | 35 | 35 |

Pairwise SNP differences were calculated based on aligned nucleotide sequences of the eight gene segments between the human-derived H9N2 virus from Case 5 (A/human/Henan/JPZ002/2025/H9N2) and genetically related environmental H9N2 viruses detected in exposure-associated environments investigated for Case 5 (A/environment/Henan/JPZ026/027/028/029/2025/H9N2). Alignment gaps were excluded from the calculation.

**Supplementary Table 6 Comprehensive amino acid substitutions and molecular markers of H9N2 influenza viruses (n = 39)**

| **Gene** | **Position** | **Mutation** | **Frequency (%)** | **No.** | **Functional annotation** |
| --- | --- | --- | --- | --- | --- |
| PB2 | 9 | D9N | 0.00 (0/39) | 0 | Virulence (mice) |
|  | 89 | L89V | 100 (39/39) | 39 | Polymerase activity / virulence |
|  | 271 | T271A | 0.00 (0/39) | 0 | Polymerase activity (compensatory) |
|  | 309 | G309D | 100 (39/39) | 39 | Polymerase activity / virulence |
|  | 339 | T339K | 100 (39/39) | 39 | Polymerase activity / virulence |
|  | 477 | R477G | 100 (39/39) | 39 | Polymerase activity / virulence |
|  | 495 | I495V | 100 (39/39) | 39 | Polymerase activity / virulence |
|  | 588 | A588/IV | 100 (39/39) | 39 | Host adaptation |
|  | 591 | Q591R | 2.56 (1/39) | 1 | Host adaptation |
|  | 627 | E627K/V | 43.59 (17/39) | 17 | Host adaptation |
|  | 631 | M631L | 0.00 (0/39) | 0 | Polymerase activity |
|  | 701 | D701N | 0.00 (0/39) | 0 | Host adaptation |
| PB1 | 3 | D3V | 100 (39/39) | 39 | Virulence |
|  | 368 | I368V | 76.92 (30/39) | 30 | Transmission potential |
|  | 622 | D622G | 100 (39/39) | 39 | Polymerase activity |
| PA | 37 | S37A | 0.00 (0/39) | 0 | Polymerase activity |
|  | 105 | F105L | 0.00 (0/39) | 0 | Drug resistance (baloxavir) |
|  | 199 | E199D | 0.00 (0/39) | 0 | Drug resistance (baloxavir) |
|  | 356 | K356R | 100 (39/39) | 39 | Virulence / replication |
|  | 383 | N383D | 100 (39/39) | 39 | Polymerase activity |
|  | 550 | I550L | 100 (39/39) | 39 | Virulence / replication |
| HA | 226 | Q226L | 100 (39/39) | 39 | Receptor binding (α2,6) |
|  | 228 | G228S | 0.00 (0/39) | 0 | Receptor binding |
|  | 264 | Y264H | 100 (39/39) | 39 | Antigenic variation |
|  | Cleavage site | PSRSSR↓GLF | 100 (39/39) | 39 | Low pathogenicity motif |
| NP | 52 | Y52N/H/Q | 100 (39/39) | 39 | Immune evasion |
|  | 105 | M105A/V | 100 (39/39) | 39 | Virulence |
|  | 184 | A184K | 100 (39/39) | 39 | Virulence |
|  | 210 | E210D | 0.00 (0/39) | 0 | Polymerase activity |
|  | 283 | L283P | 0.00 (0/39) | 0 | Immune evasion |
|  | 313 | F313Y/V | 0.00 (0/39) | 0 | Immune evasion |
|  | 357 | Q357K | 0.00 (0/39) | 0 | Host adaptation |
| NA (N2 numbering) | 63–65 | Deletion | 100 (39/39) | 39 | Host adaptation |
|  | 274 | H274Y | 0.00 (0/39) | 0 | Drug resistance |
|  | 292 | R292K | 0.00 (0/39) | 0 | Drug resistance |
| M1 | 15 | V15I | 100 (39/39) | 39 | Virulence |
|  | 30 | N30D | 100 (39/39) | 39 | Virulence |
|  | 43 | I43M | 100 (39/39) | 39 | Virulence |
|  | 166 | A166V | 0.00 (0/39) | 0 | Host adaptation |
|  | 215 | T215A | 100 (39/39) | 39 | Virulence |
| M2 | 31 | S31N | 100 (39/39) | 39 | Drug resistance |
| NS1 | 42 | P42S | 100 (39/39) | 39 | Virulence |
|  | 103 | L103F | 0.00 (0/39) | 0 | Immune evasion |
|  | 106 | I106M | 0.00 (0/39) | 0 | Immune evasion |
